# Imbalanced pattern completion vs. separation in cognitive disease: network simulations of synaptic pathologies predict a personalized therapeutics strategy

**DOI:** 10.1186/1471-2202-11-96

**Published:** 2010-08-13

**Authors:** Jesse E Hanson, Daniel V Madison

**Affiliations:** 1 Department of Molecular and Cellular Physiology, Stanford University School of Medicine, Stanford, CA 94305, USA; 2 Genentech, Inc., South San Francisco, California 94080, USA

## Abstract

**Background:**

Diverse Mouse genetic models of neurodevelopmental, neuropsychiatric, and neurodegenerative causes of impaired cognition exhibit at least four convergent points of synaptic malfunction: 1) Strength of long-term potentiation (LTP), 2) Strength of long-term depression (LTD), 3) Relative inhibition levels (Inhibition), and 4) Excitatory connectivity levels (Connectivity).

**Results:**

To test the hypothesis that pathological increases or decreases in these synaptic properties could underlie imbalances at the level of basic neural network function, we explored each type of malfunction in a simulation of autoassociative memory. These network simulations revealed that one impact of impairments or excesses in each of these synaptic properties is to shift the trade-off between pattern separation and pattern completion performance during memory storage and recall. Each type of synaptic pathology either pushed the network balance towards intolerable error in pattern separation or intolerable error in pattern completion. Imbalances caused by pathological impairments or excesses in LTP, LTD, inhibition, or connectivity, could all be exacerbated, or rescued, by the simultaneous modulation of any of the other three synaptic properties.

**Conclusions:**

Because appropriate modulation of any of the synaptic properties could help re-balance network function, regardless of the origins of the imbalance, we propose a new strategy of personalized cognitive therapeutics guided by assay of pattern completion vs. pattern separation function. Simulated examples and testable predictions of this theorized approach to cognitive therapeutics are presented.

## Background

Impaired cognition occurs in many different neurodevelopmental, neuropsychiatric, and neurodegenerative diseases. The identification of numerous disease-linked gene mutations has led to the creation of various transgenic mouse models that replicate the phenotypes of human patients, especially learning and memory impairments. Our analysis begins with a review of existing neurophysiological and neuroanatomical experiments in diverse genetic models of impaired cognition that include memory deficits. This synthesis of the literature highlights four properties of synaptic or neural network function that are commonly altered in these conditions: 1) Strength of LTP, 2) Strength of LTD, 3) Relative inhibition, and 4) Connectivity levels (**Table **1).

**Table 1:** Key Synaptic Phenotypes in Mouse Models of Diseased Cognition

Disease Model	LTP	LTD	Inhibition	Connectivity
Neurodevelopmental				

**Angelman synd.**	Decreased [40,46]			Decreased [47]
*Ube3A *KO				

**Down synd.**	Decreased [48-50]	Increased [51]	Increased [24,49]	Decreased/Increased* [24,52,53]
Trisomy				

**Fragile × synd.**	Decreased/Increased [54-56]	Increased [57]	Decreased [58-60]	Decreased/Increased* [25,27,28]
*Fmr1 *KO				

**FRAXE synd.**	Increased [61]			
*Fmr2 *KO				

**Neurofibromat.**	Decreased [62-64]		Increased [62,63]	
*Nf1 *het				

**Rett synd.**	Decreased [65,66]	Decreased [65]	Increased [67]	Decreased [68,69]
*Mecp2 *KO				

**Tuberous Scler.**	Decreased [70]	Decreased [70]		Decreased [71,72]
*Tsc1 *KO				
*Tsc2 *KO (rat)				

**Various XLMR**	Decreased [73,74]	Decreased [75]		
*Ophn1 *KO				
*Pak3 *KO				
*Gdi1 *KO				

Neuropsychiatric				

**Schizophrenia**	Decreased [76,77]	Decreased [78]	Decreased [78]	Decreased [76,79-81]
*Disc1 *mut				
*Reelin *het				
22q11 del				

Neurodegenerative				

**ALS**	Increased [82]			Decreased [83]
*Sod1 *mut				

**Alzheimer's**	Decreased [30,32,84-86]		Increased [30,32]	Decreased/Increased* [26,84,87-89]
*App *mut				
*Ps1*/*Ps2 *KO				
*App*/*Ps1 *mut				

**Huntington's**	Decreased [38,90,91]	Decreased/Increased [92,93]		
*Htt *mut				

**Parkinson's.**	Decreased/Increased [94,95]	Decreased [94]		
*Dj-1 *KO				
Parkin KO				

**SCA**	Decreased [96,97]			Decreased [98]
*Sca1 *mut				
*Fgf14 *KO				

The first column lists neurological conditions associated with impaired cognition, along with corresponding diseased-linked mutations that have been modeled in mice. The remaining columns list reported alterations to LTP, LTD, synaptic inhibition, and connectivity in each group of mouse models. Asterisks indicate reports of increased connectivity that are accompanied by concomitant decreases in connectivity in different neuronal subpopulations. As well as direct physiological measurement of synaptic connectivity, indirect findings of altered dendritic spine density or axonal projections were considered indications of altered connectivity. While the connections within CA3 are the primary focus of our simulations, studies of synapses throughout the hippocampus are listed to allow a comprehensive comparison of existing data. When no hippocampal data was available, studies of other cortical neuron populations were included.

**Figure 1:**
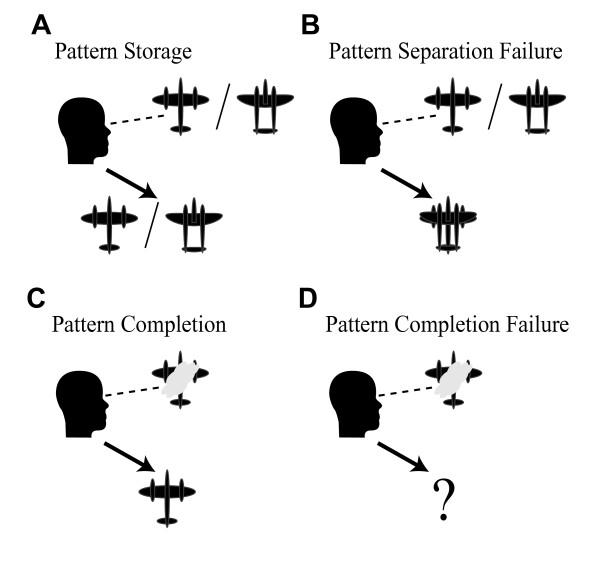
**Autoassociative Memory: Pattern Storage, Completion, and Separation**. Autoassociative memory involves the rapid automatic generation of internal representations of sensory stimuli. Key functions of autoassociative memory are depicted with stimulus patterns consisting of visualized airplane silhouettes. A) Pattern storage includes the ability to simultaneously store representations of multiple stimuli, such as similar yet distinct airplane silhouettes. B) Pattern separation fails during pattern storage when multiple distinct stimuli cannot be simultaneously stored without interference. C) Pattern completion is the ability to recall a stored representation when cued by a partial or degraded observation of the stimulus, such as when an airplane is obscured behind a cloud. D) Pattern completion fails when the degraded stimulus is insufficient to result in recall of the entire stored representation.

To explore potential network level impacts of these four convergent points of synaptic pathology, we examined the performance of a neural network simulation of autoassociative memory while varying the strength of each synaptic property. Associative memory requires binding separate elements of a sensory experience into a single memory that can be later recalled in its entirety, even when cued by only some of the original elements. Autoassociation is the ability of neural networks to perform associative memory without any external guidance, via changes in synaptic strengths caused by neuronal activity. Autoassociative memory in area CA3 of the hippocampus in particular, is perhaps the best example of a convergence of theoretical predictions [1-3] and experimental evidence [4-8] for how neural networks store memories, and is thought to represent a basic process essential to the learning capabilities of interconnected brain networks [9,10]. Two key features supported by autoassociative memory are pattern separation, and pattern completion. Pattern separation is the ability to keep distinct memory patterns separate during storage (**Fig. **1A,B), while pattern completion is the ability to recall an entire stored memory pattern in response to a degraded or partial observation of elements of the stored pattern (**Fig. **1C,D). Analytical models of autoassociation have described a trade-off between pattern completion and separation functions that is influenced by the strength of LTP and LTD [11]. Previous work also indicates that autoassociative network capacity is dependent on both excitatory connectivity levels and the properties of synaptic inhibition [12,13]. Therefore, we hypothesize that the cognitive diseased-linked synaptic pathologies of LTP, LTD, inhibition, and connectivity should all converge in affecting memory performance by shifting the underlying trade-off between pattern completion and pattern separation. In particular we predict that while the net effect of some constellations of pathologies will be severe deficits in pattern separation, other constellations of pathologies will impair memory performance due to severely impaired pattern completion. Moreover, regardless of the underlying deficits, appropriate manipulation of any of the four synaptic functions could help correct imbalanced autoassociative function. These hypotheses of pathology and therapeutics were tested using network simulations based on current concepts of autoassociative function.

## Results

### Diverse disease models exhibit convergent synaptic and circuit alterations

Examples of neurodevelopmental diseases that can include memory deficits, where causative genes have been identified and mouse models have been created, include Angelman syndrome, Down syndrome, Fragile × syndrome, FRAXE Syndrome, Rett Syndrome, Neurofibromatosis, Tuberous Sclerosis, and various X-linked Mental Retardations (XLMR) (Table 1, top). Transgenic mouse models have also been created that are relevant to neuropsychiatric conditions including schizophrenia, a disease where memory impairment is an important endophenotype (Table 1, middle). In addition to Alzheimer's disease, other neurodegenerative diseases often more noted for their hallmark motor symptoms, also feature important cognitive phenotypes, and mouse models of neurodegenerative conditions with memory alterations include Amyotrophic Lateral Sclerosis (ALS), Huntington's disease, Parkinson's disease, and Spinocerebellar Ataxia (SCA) (Table 1, bottom). Together, these diverse mouse models provide a comparative window into potential substrates of memory impairment. In particular reoccurring points of pathological changes include: 1) Strength of LTP, 2) Strength of LTD, 3) Relative inhibition, and 4) Connectivity levels.

### Neural Network Simulation of Autoassociation

**Figure 2:**
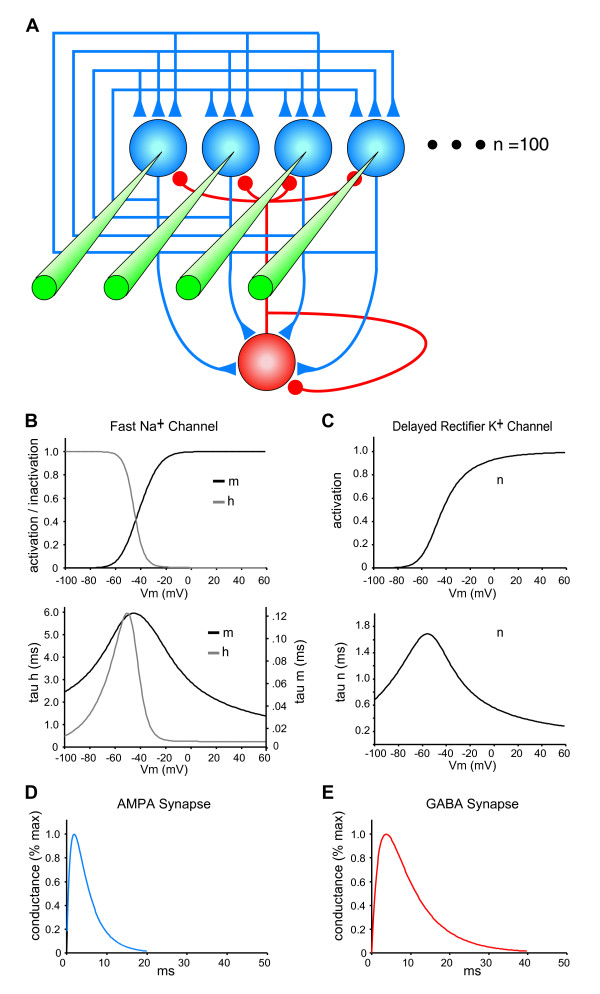
**Autoassociative Network Model**. (A) The architecture of the 100 neuron network is illustrated with four exemplar excitatory neurons (blue). The recurrent interconnections (associational synapses) in a network with full connectivity are shown with blue triangles. Excitatory synapses are also made from each excitatory neuron onto a single interneuron that represents the total inhibition of the network (red neuron). Feedback inhibitory connections made by the interneuron onto each excitatory neuron are shown with red circles. Feedback inhibition onto the interneuron is represented using an autaptic connection. Depicted in green is the input to each neuron used to activate a neuron as part of a stimulus pattern. Each biophysically reduced model neuron had membrane properties based on a model of rhythm generation in the hippocampus [15] (see methods). (B) Voltage-gated Fast Na^+ ^conductance steady state activation and inactivation (top) and time constants (bottom) of the m and h gating variables are shown. (C) Voltage-gated Delayed Rectifier K^+ ^conductance steady state activation (top) and time constant (bottom) of the n gating variable is shown. (D) AMPA conductance time courses are shown. (E) GABA conductance time courses are shown.

**Figure 3:**
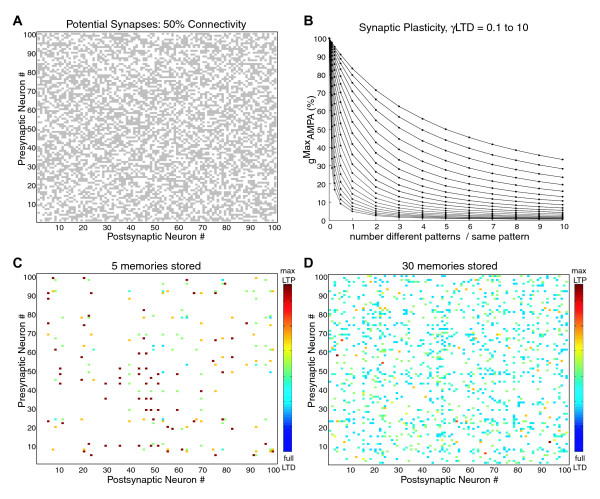
**Synaptic Connectivity and Plasticity**. A) Example matrix of potential synaptic interconnections between the 100 excitatory neurons in a network with 50% connectivity. Gray squares show possible (silent) synapses, and white squares indicate the absence of anatomical synapses, as a function of postsynaptic (x axis) and presynaptic (y axis) neuron identity. B) Synaptic plasticity was implemented with the parameter, g^Max^AMPA specifying the strength of a fully potentiated synapse resulting from co-activation of a presynaptic and postsynaptic neuron during storage of a stimulus pattern. The parameter γ^LTD ^determined the strength of depression resulting from asynchronous activation of a presynaptic and postsynaptic neuron during different stimulus patterns within a set of stored memories. The resulting profile of plasticity for different numbers of asynchronous activations per synchronous activation is show for γ^LTD ^values logarithmically spaced between 0.1 (top series) and 10 (bottom series). C) The synaptic strength matrix of the example network with 50% connectivity is shown after 5 patterns have been stored, using an intermediate value of γ^LTD^. Un-strengthened (silent) synapses are white, and the strengths of synapses ranging from fully potentiated to strongly depressed are illustrated with the color scale from red to blue. D) The synaptic strength matrix of the example network is shown after 30 patterns have been stored. Note that while more synaptic connections have been potentiated, accumulation of overlap between the patterns has resulted in stronger average depression in the network compared to the storage of 5 patterns.

To explore the impact of synaptic alterations on autoassociative functions, we used a neurobiologically realistic, reduced network model of autoassociation that allowed modulation of each of the four points of synaptic pathology. This model was based on a published model of hippocampal area CA3, in which the subpopulations of neurons that are active during distinct gamma cycles are the substrates of memory storage and recall [13,14]. While replicating the concept of this previous model, our implementation used simplified biophysically realistic neurons with properties taken from a model of rhythm generation in the hippocampus [15] (see methods, **Fig. **2). It should be noted that the model used here resembles previous models that use biophysically detailed individual neurons within a simplified neural circuit [16,17], rather than models with more complex network interactions and more sophisticated memory function, but simpler single neuron representations [18-20]. All of our simulations focused on networks of 100 excitatory neurons with feedback inhibition, and each pattern to be stored and recalled consisted of the activation of a set of 10 individual neurons (**Fig. **2,3). Each of the four synaptic phenotypes observed to be increased or decreased in mouse models of cognitive impairment corresponded directly to variables within the simulations as follows:

#### Connectivity

The connectivity level of the recurrent excitatory synapses was set to a given value (for example 50%; **Fig. **3A), which was implemented by constraining the average number of randomly selected postsynaptic target neurons for each presynaptic neuron.

#### LTP and LTD

In the absence of any stored memories, all potential synaptic connections were silent with no AMPA receptor (AMPAR) conductance. LTP was implemented by potentiating synapses between presynaptic and postsynaptic neurons that both fired action potentials within the time window of a single gamma cycle during pattern storage. The strength of LTP was defined by the maximal excitatory synaptic conductance variable, g^Max^AMPA. LTD was implemented by depressing synapses between presynaptic neurons and postsynaptic neurons that were active during storage of different stimulus patterns within a set of simultaneously stored patterns. The parameter, γ^LTD^, defined the strength of LTD (see methods, **Fig **3B). The result of these plasticity mechanisms is that as larger numbers of patterns are simultaneously stored in the network, more silent synapses are potentiated, and more of those potentiated synapses are also reduced in strength by LTD due to accumulated overlap of the stimulus patterns (**Fig. **3C,D).

#### Relative Inhibition

The strength of the feedback inhibitory conductance, gGABA, received by each pyramidal neuron was set relative to the average maximal excitatory conductance received by excitatory neurons, as defined by the Inhibition Ratio variable (see methods). Thus, the strength of LTP, LTD, connectivity, and inhibition could all be varied to simulate pathological conditions or therapeutic modulation via manipulation of single variables.

### Measuring Pattern Completion and Pattern Separation

**Figure 4:**
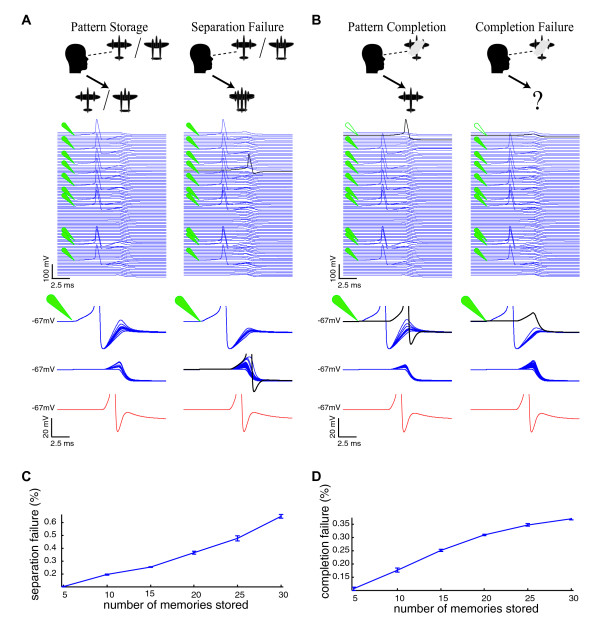
**Simulated Pattern Separation and Completion**. Simulated autoassociative functions are illustrated (after deAlmeida et al., 2007). A) The simulated membrane potentials of all 100 excitatory neurons (blue) are illustrated (top) for the time window corresponding to the peak of a single gamma cycle. Green symbols indicate the 10 neurons activated during storage of a stimulus pattern. Also shown (bottom) on an expanded voltage scale are the overlaid near-threshold membrane potentials of the 10 neurons participating in the stimulus pattern (green symbol), the other 90 excitatory neurons in the network, and the inhibitory neuron (red). Pattern separation is successful when, as new memories are stored, only the 10 stimulated neurons fire action potentials, without extraneous firing in the network during the cycle. Pattern separation fails when extraneous firing in one or more neurons outside of the stimulus occurs during pattern storage (black). B) Pattern completion is tested by stimulating only 9 of the 10 neurons in a stored pattern. The neuron that is part of a pattern but not stimulated during the degraded stimulus presentation is depicted with an open green symbol. Pattern completion is successful when synaptic input from the other 9 neurons is able to elicit firing in this neuron (black) within the restricted time window shown, corresponding to a single gamma cycle. Failure of pattern completion occurs when the degraded stimulus is unable to induce firing in the un-stimulated neuron. C) The rate of pattern separation errors is shown as a function of the number of stored patterns for an example set of network parameters. D) The rate of pattern completion errors as a function of the number stored patterns is shown.

Sensory stimulus patterns representing distinct memories each consisted of the direct activation of 10 of the 100 neurons in the network. Sets of stimulus patterns, equivalent to a list of multiple memories to be maintained simultaneously, were selected at random with larger sets having greater average overlap between the stimulus patterns. Sets of patterns were stored in the network by calculating and applying the synaptic plasticity resulting from the storage of an entire set of stimulus patterns (for example a set of 5 patterns each consisting of the activation of 10 neurons). Since interleaved learning was assumed, the dynamics of sequential storage of different patterns were not modeled [13]. Pattern separation was tested by activating the 10 neurons of each stored pattern, and evaluating any spurious firing in the remainder of the network during the time window corresponding to the peak of a single gamma cycle (**Fig. **4A). Activation of one or more neurons not participating in a pattern was considered separation failure for that pattern. Pattern completion was evaluated by activating 9 out of 10 neurons in a stored pattern, and checking for evoked firing of the 10^th ^neuron of that pattern within the same gamma cycle (**Fig. **4B). Lack of firing of the 10th neuron during that gamma cycle was considered pattern completion failure. This reduced network of 100 neurons using stimuli consisting of activation 10 neurons was sufficiently complex to model pattern completion and separation analogous to that depicted in **Fig. **1. Rates of pattern completion and pattern separation errors per memory pattern as a function of the number of simultaneously stored stimulus patterns were measured for each set of simulation conditions (see methods, **Fig. **4C,D). Initial simulations revealed that both pattern completion and pattern separation error rates increased as the number of simultaneously stored memories increased.

### Wild-type Synaptic Properties

While biologically plausible values of each synaptic property serve as a good starting point for the baseline model, the necessary simplifications of the model make it difficult to predict exact wild-type values of synaptic properties. For example, relative inhibition is specified using the ratio of GABA_A _receptor (GABA_A_R) to AMPAR conductance in each spatially reduced neuron. However the dynamics of synaptic interaction in spatially complex neurons, can enhance the ability of inhibition to oppose excitation [21,22], suggesting amplified GABA_A_R to AMPAR conductance ratios may be needed in the simplified simulation. Because of such considerations, we decided to determine wild-type network parameters via an empirical assessment of storage and recall performance. To avoid focusing on a non-unique set of optimal parameters we explored network performance over a broad range of parameter space by creating a database with 960,000 combinations of parameter values (g^Max^AMPA, γLTD, Relative Inhibition, and Connectivity %) and memory storage conditions (see methods). While different metrics could be used to assess network performance, we defined optimal networks based on the maximal error rate, which was calculated as the greater of pattern completion or pattern separation error rates. Using this measure, pattern completion and pattern separation errors were equal in their ability to limit network performance.

**Figure 5:**
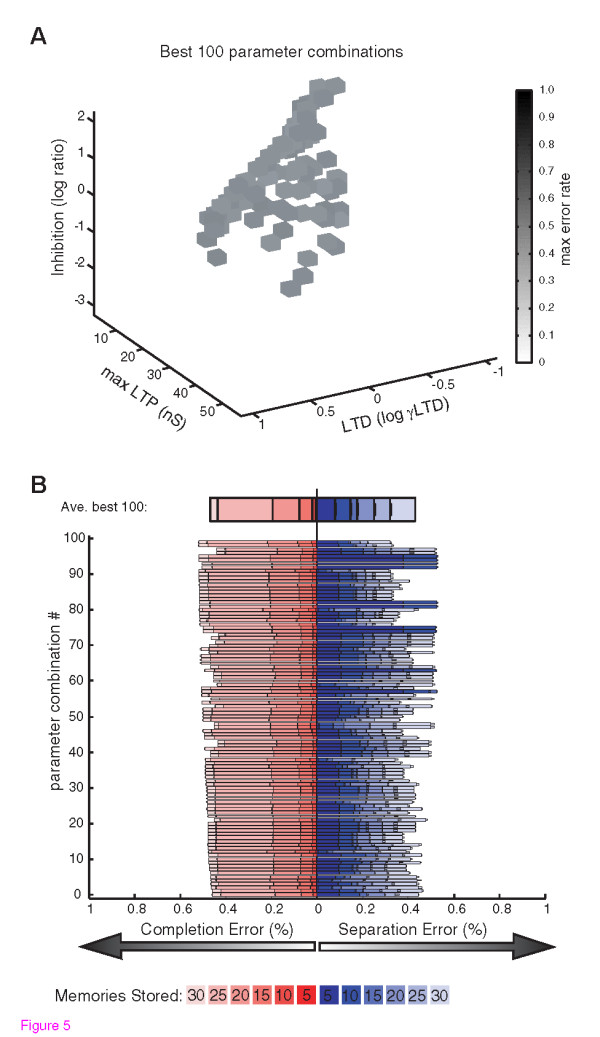
**Multiple Synaptic Parameter Permutations Support Optimal Balanced Function**. A) Optimal balanced networks were found by searching for parameter combinations that produced the lowest maximal error (the higher error in either pattern separation or pattern completion) during the storage of 30 patterns. The 100 best combinations of the parameters defining LTP, LTD, and inhibition, are shown with connectivity constrained to 50%. The maximal error rate is shown in grayscale. The ability of parameter combinations spanning a broad range or parameter space to achieve similar performance is evident. B) The balances of pattern completion errors (increasing from 0 at the center to the left) and pattern separation errors (increasing from 0 at the center to the right) are shown for the 100 best parameter combinations and for their average. Different shades of red and blue are overlaid to show the error rates for different numbers of stored patterns for pattern completion, and pattern separation, respectively.

In the initial evaluation of optimal parameter combinations, 100% connectivity levels were always found to allow the best performing networks, although this required extremely low values of LTP, and extremely high values of LTD and inhibition. However, anatomically-based estimates of *in vivo *connectivity within CA3 are more sparse, with estimates ranging from very low up to 50% [13,23].The higher value of 50% connectivity is consistent with physiological observations of ~50% in cultured brain slices that have re-grown CA3 connections severed during slice preparation, but maintain the total synaptic input levels seen in acutely prepared slices [24,25]. Supporting an upper limit on total connectivity, pathological increases in connectivity within neuronal subpopulations are often accompanied by decreased connectivity in other subpopulations [24-26]. Therefore, to maintain biological realism and minimize neuronal number in the simulations, while defining wild-type networks, we searched the parameter space of the other three synaptic properties, while constraining wild-type connectivity levels to 50%. Optimal balanced networks were found by searching for parameter combinations that produced the lowest maximal error (the higher error in either pattern separation or pattern completion). This analysis revealed that a relatively broad contour of LTP, LTD, and inhibition, parameter space could support optimal balanced network function (**Fig. **5A). Thus, the illustrated 100 best parameter combinations reflect a diversity of approximately equally well-balanced wild-type networks, perhaps analogous to variability in the *in vivo *networks of healthy individuals (**Fig. **5B). Accordingly, in assessing the impact of simulated synaptic pathologies, we considered the average effect of pathologies to each of these 100 optimal 'wild-type' parameter combinations.

### Pattern Separation and Completion with Single Synaptic Pathologies

**Figure 6:**
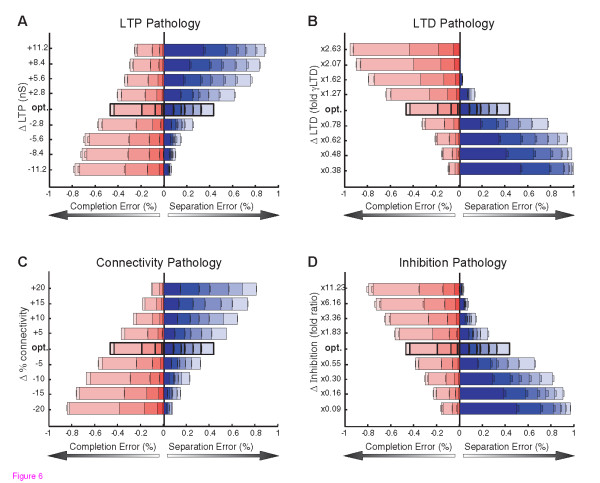
**Each Synaptic Pathology Alters the Trade-Off between Completion and Separation**. The average performance of the 100 best parameter combinations illustrated in Figure 5, were used to represent optimal autoassociative performance. To simulate pathological changes in each synaptic property, parameter values were increased or decreased by various degrees in each of the 100 optimal networks, and the error rates in pattern completion and separation were re-assessed. A) Changes in the strength of LTP were implemented by increases or decreases in g^max^AMPA, relative to optimal values (opt, y axis). B) Changes in the strength of LTD were implemented by multiplying optimal γ^LTD ^values by different amounts. C) Connectivity levels were increased or decreased relative to the optimal level (50%). D) Changes in relative inhibition were implemented by multiplying optimal values by different amounts. Each simulated pathology shifted the trade-off between completion and separation, with opposite effects when the synaptic properties were increased or decreased.

To test the isolated impact of each synaptic pathology on memory performance, we analyzed pattern completion and separation error rates as LTP, LTD, inhibition, and connectivity, were each varied relative to the optimal wild-type networks (**Fig. **6). These simulations revealed a striking trade-off between pattern completion errors and pattern separation errors. In particular, increasing or decreasing the value of any given synaptic function could decrease one type of error, but at the expense of increasing the other. High connectivity, strong LTP, weak LTD, or weak inhibition, all reduced pattern completion errors, but did so at the expense of increased errors in pattern separation. Conversely, low connectivity, weak LTP, strong LTD, and strong inhibition could all reduce pattern separation errors, but at the expense of increased errors in pattern completion (**Fig. **6). The observed extreme error rates in either pattern completion or separation, resulting from the synaptic pathologies are predicted to be sufficient to impair performance on standard memory tasks. On one hand, low rates of separation errors during storage are irrelevant in the face of intolerable rates of completion failure during recall. Conversely, low rates of completion error during recall would be masked by intolerable rates of separation failure during storage. Thus, imbalanced networks with a bias towards either pattern separation or pattern completion are potential substrates of the learning and memory impairments observed in neurological disease.

### Interaction Between Multiple Synaptic Alterations

**Figure 7:**
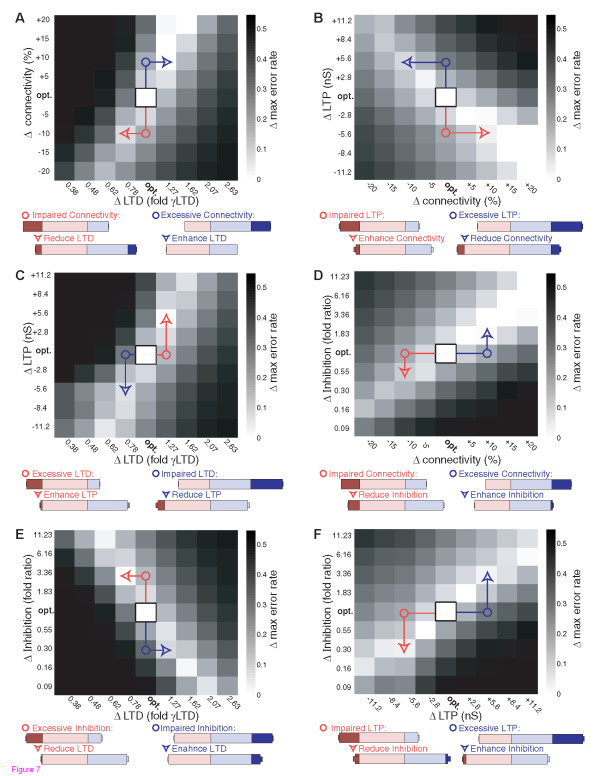
**Concomitant Synaptic Modulations Can Exacerbate or Rescue Imbalances**. A-F) Changes to optimal network balance are shown for each permutation of simultaneous changes in two of the four synaptic properties. Grayscale values illustrate increases in max error rate relative to the optimal networks. Optimal networks are central in the 2D plots, bordered with a black box. Increasing max error rates resulting from alterations to single parameters are seen in the vertical or horizontal deviations from the optimal networks, while the remainder of each plot shows the effects of concomitant alterations to both parameters. Examples of pathological shifts towards intolerable pattern completion error (separation performance bias, red circles), or pathological shifts towards intolerable pattern separation error (completion performance bias, blue circles) are highlighted for individual synaptic pathologies. Error rates for these highlighted data points are shown, with pale red and blue bars illustrating completion and separation error rates within tolerable limits defined by optimal network performance. Completion or separation error rates exceeding tolerable limits are illustrated with dark red or dark blue, respectively. For each highlighted pathology, examples of therapeutic shifts towards more tolerable error rates that could be achieved by manipulating the other synaptic property are shown with red arrowheads (amelioration of separation biases) or blue arrowheads (amelioration of completion biases). Note that while increases in connectivity are included for completeness, that upper limits are likely biologically constrained, limiting the plausibility of the low error regions of parameter space seen with high connectivity (see text).

Because the synaptic pathologies all shifted the trade-off between completion and separation, we examined the interaction of multiple simultaneous pathologies (Fig. 7). This analysis of simultaneous increases or decreases in all combinations of the four synaptic properties showed that any two synaptic alterations that could both alone caused a separation or completion bias, would together cause an exacerbation of the bias. Conversely any two synaptic alterations that caused opposites shifts in network balance had the potential to at least partially offset each other. This finding of interchangeable effects of simultaneous synaptic alterations has implications for compensatory mechanisms and therapeutic re-balancing of network function. Given that intolerable levels of error in either pattern separation or pattern completion could be limiting factors in memory performance, manipulations that shift the balance so as to ameliorate extreme imbalances should be of therapeutic benefit. Specific examples of imbalances causing increased maximal error resulting from various synaptic pathologies, along with therapeutic ameliorations of these effects by manipulation of independent synaptic properties are shown in **Fig. **7. In such attempts to correct networks disrupted by one abnormal synaptic property via manipulations of independent synaptic properties, more balanced minimization of both types of error can be achieved, even if some residual increased errors relative to the optimal networks remain.

## Discussion and Conclusions

### Substrates of Imbalanced Network Performance

**Figure 8:**
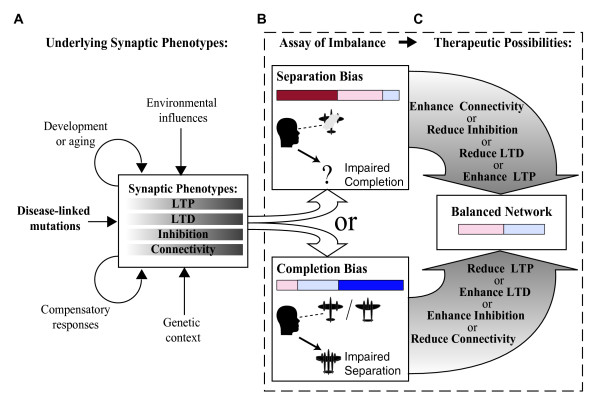
**Personalized Therapeutics Based on Assay of Network Imbalance**. A) The origins of pathological changes in the synaptic phenotypes of LTP, LTD, inhibition, and connectivity are indicated. In addition to direct effects of disease-linked mutations (**Table 1**), and other genetic or environmental factors, effects of feedback due to developmental or age-related changes, as well as homeostatic compensatory mechanisms, are indicated. B) The key prediction of the simulations is that the summed impact of any constellation of synaptic pathologies will be to imbalance network performance in one of two directions, 1) Separation bias, where despite very low pattern separation error, intolerable errors in pattern completion underlie memory impairment, or 2) Completion bias, where despite very low completion error, intolerable errors in pattern separation underlie memory impairment. C) The proposed approach of using assays of network imbalance to prescribe therapeutic targets is illustrated. Manipulations of each of the synaptic properties predicted to have therapeutic benefits are listed for each type of network imbalance (large arrows). The dashed box is to emphasize that the approach of predicting therapeutics based on assay of network imbalance is agnostic to the underlying synaptic phenotypes and their causes.

The simulation results demonstrate that increases or decreases in LTP, LTD, inhibition, or connectivity, as observed in mouse models of disease-linked mutations, can shift the balance of autoassociative function towards intolerable error in either pattern completion or pattern separation. However, in individual patients, the impacts of disease-linked mutations will likely be modulated by multiple genetic and environmental factors. For example, even when identical mutations are examined in different background mouse strains (different genetic contexts) opposite pathologies in connectivity are observed within models of Fragile × Syndrome [27,28] or AD [29]. In addition, many synaptic phenotypes develop with age, indicating that disease-linked mutations have different impacts during development and aging, perhaps in part due to induction of compensatory mechanisms [30]. The various factors that could contribute to manifestation of pathology in the four synaptic phenotypes are illustrated in **Fig. **8A. Regardless of the underlying origins, however, the ability of multiple synaptic alterations to compound or counteract each other in shifting the balance between pattern completion vs. separation errors (**Fig. **7) suggests that a large number of combinations of synaptic pathologies will have a net effect of a bias towards either pattern completion or pattern separation.

To intuitively understand the basis of the interactions between the different points of synaptic pathology it is important to appreciate that the underlying substrates of successful pattern completion (and failed separation) necessarily converge at action potential generation, while essential to pattern separation (and failed completion) is lack of inappropriate spiking. Thus while the effects of LTP, LTD, inhibition and connectivity levels all impinge on the ability of synaptic inputs to drive output spiking, other perturbations that also effect input-output coupling will also alter the balance between completion and separation. For example, while beyond the scope of the present analysis, neuromodulatory influences that alter intrinsic excitability are also aberrant in disease states and are expected to alter pattern separation and completion functions [19,31].

### Theory of Personalized Therapeutics

That the four examined points of synaptic pathology all further converge in causing one of two distinct network imbalances provides a potential point of therapeutic intervention (**Fig. **8B). In particular, a pattern completion bias predicts one direction of therapeutic manipulation for each synaptic property, while a separation bias predicts therapeutic value for the opposite directions of manipulation (**Fig. **8C). If pattern completion vs. pattern separation performance were to be extensively evaluated in cognitive disease, two general possibilities exist: 1) Some causes of disease will consistently involve a separation bias, while other causes will always involve a completion bias, 2) Even within the same disease, the complex interaction of genetics and environment with disease progression will result in some patients with a completion bias and others with a separation bias.

In the case of uniform biases within a disease this model is useful in predicting cross-therapeutic value of drugs with different targets. All of the reported synaptic phenotypes in AD would push network balance towards a separation bias predicting unambiguous therapeutic targets (**Fig. **8C**, top**), which is supported by the observation that decreasing inhibition rescues memory performance in mice modeling AD [32]. However, in other disease models, phenotypes causing opposite biases co-exist (**Table **1), and the appropriate therapeutic targets are thus unclear because they will depend on the relative magnitude of the different pathologies. For example Down syndrome model mice exhibit AD-like phenotypes of reduced LTP, enhanced LTD, and enhanced inhibition, but also exhibit enhanced recurrent connectivity that would favor a completion bias. Insight comes from the observation that as with AD model mice, GABA_A_R antagonists can rescue impaired memory in the Down syndrome model mice [33,34] which is consistent with a net pattern separation bias. Therefore, in addition to drugs decreasing inhibition, the theory predicts that drugs enhancing LTP, and connectivity, and/or reducing LTD should also be of therapeutic value in Down syndrome (**Fig. **8C, **top; Table **2). In another example, schizophrenia models exhibit phenotypes both supporting a separation bias (impaired LTP, and decreased connectivity) and supporting a completion bias (impaired LTD, and decreased inhibition). In this case, that enhancement of GABA_A_R function can rescue impaired memory in Schizophrenia patients[35,36], suggests a net completion bias dominates. Therefore, along with positive modulators of GABA_A_Rs, drugs that would selectively reduce LTP, enhance LTD, and/or decrease connectivity are also predicted to be valuable for treating cognitive deficits in Schizophrenia (**Fig. **8C, **bottom; Table **2). Further examples of extrapolating cross-therapeutic efficacy come from mouse models that have been treated with manipulations that can increase LTP. In particular, enhancements of LTP by PAK inhibition in Fragile × Syndrome model mice [37], by BDNF increases in Huntington's disease model mice [38,39], or by reduction of αCaMKII inhibitory phosphorylation in Angelman syndrome model mice [40], are all accompanied by rescued memory performance. These findings are consistent with an underlying pattern separation bias in these conditions, and predict efficacy of the corresponding list of therapeutic targets (**Fig. **8C, **top; Table **2).

**Table 2:** Example Cross-Therapeutic Predictions for Overall Averages of Disease Populations

Disease or model	Memory rescue observed with	Other predicted targets
**Alzheimer's mouse**	↓ Inhibition [32-34]	↑ LTP, ↑ connectivity, ↓ LTD
**Down synd. mouse**		

**Human Schizophrenia**	↑ Inhibition [35,36]	↓ LTP, ↓ connectivity, ↑ LTD

**Angelman mouse**		
**Fragile × mouse**	↑ LTP [37-40]	↓ Inhibition, ↓ LTD, ↑ connectivity
**Huntington's mouse**		

**Figure 9:**
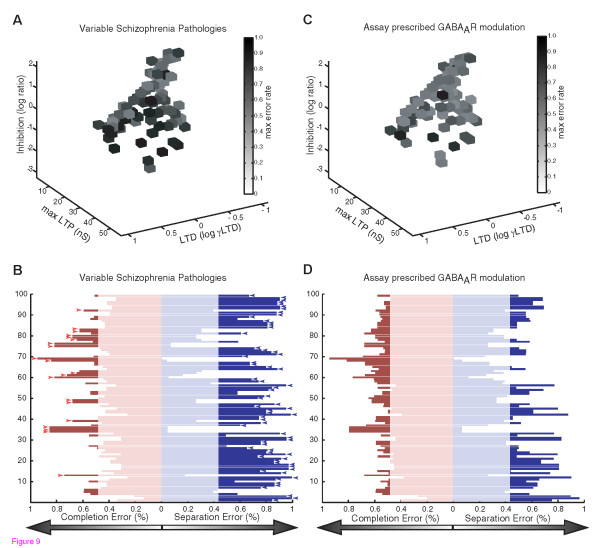
**Example of Personalized Therapeutics in Groups with Heterogeneous Pathologies**. The personalized therapeutics approach is illustrated using the example of a group of patients with pathological decreases in LTP, LTD, Inhibition, and Connectivity, as have been reported in various models related to Schizophrenia, for example (Table 1). To provide analogy with the heterogeneous genetic and environmental factors seen in human patients, the 100 optimal neural networks were each perturbed with randomly varying degrees of decreases in each synaptic property, such that an average pattern completion bias was seen in the population, consistent with average rescue of memory impairment by increased inhibition in populations of Schizophrenia patients (see Discussion). A) The maximal error in each of the pathologically perturbed networks is illustrated. For direct comparison with the wild-type networks in Figure 5, values of LTP, LTD, and inhibition are illustrated, while connectivity rates are not shown. B) The pattern completion and separation error rates with 30 patterns stored are illustrated for each of the individual pathological networks. Completion or separation error rates exceeding average tolerable limits of wild-type networks are illustrated with dark red or dark blue, respectively. Red arrowheads indicate assayed pattern separation biases, where GABA_A_R antagonists would be prescribed, while blue error heads indicate pattern completion biases, where GABA_A_R positive modulators would be prescribed (majority of individuals). C) The maximal error rates in each network following therapeutic correction as indicated in panel B, is shown. D) The pattern completion and separation error rates following therapeutic improvement with drugs targeting inhibition are shown for the individual networks.

The first column lists disease conditions or mouse models where therapeutic rescue of memory has been observed. The second column lists the synaptic property that was targeted or observed to be altered by the treatment (see discussion for details). The third column lists other predicted targets that should function to rebalance memory performance by altering the pattern completion vs. pattern separation bias with the same polarity as the observed effective treatment (see Figure 8). Note that while differential involvement of compensatory mechanism, genetic context, etc., between individuals is expected to result in heterogeneity within a given disease group, that significant improvements were seen on averaged measures of memory function in these studies supports these coarse predictions of therapeutic efficacy within groups. The potential need for personalized assay of completion vs. separation function is illustrated in Figure 9.

In the case of non-uniform biases within a disease group, assaying pattern completion vs. separation would be especially valuable for prescribing personalized therapeutics. Moreover, this approach could allow therapeutic prescription even in patients with learning and memory impairments with unknown etiologies and no clear disease diagnosis. **Fig. **9 illustrates this personalized therapeutics approach using the example of a heterogeneous group of patients with the type of pathologies seen in Schizophrenia models (**Table **1). Genetic and environmental diversity is represented by starting with the 100 optimal networks with different underlying combinations of synaptic properties, and by implementing varying degrees of the pathological decreases in each synaptic pathology as observed in mouse models of Schizophrenia. While the average autoassociative function in the simulated Schizophrenia cohort reflects a completion bias, high variability between the individuals is evident (**Fig. **9B). Simply treating the entire group with manipulations such as enhanced inhibition, would results in some therapeutic rebalancing of the group on average, consistent with the findings that GABA_A_R positive modulators can improve memory performance in schizophrenia patients. However, assay of individual autoassociative biases in each simulated patient leads to the identification of both patients with completion biases that would especially benefit from manipulations including increased inhibition, as well as a some patients with separation biases who would benefit from opposite manipulations including decreased inhibition (**Fig**. 9C,D).

How pattern separation and pattern completion deficits in neural networks will read out in indirect behavioral measurements such as tests of prospective interference may be complicated, but could be determined by experiments correlating behavior and read-outs of neural network separation/completion function. However, the promise for implementing such an approach of assay-based therapeutic prescription is good, since non-invasive touchscreen-based memory tests already exist, including explicit measurement of pattern separation function, in both mouse models [41] and human patients [42,43]. While higher-order processing strategies could confound behavioral read-outs of basic network functions [42], direct assay of autoassociative function can be performed in rodent models using recordings of neuronal ensemble activity [4-6], and has been demonstrated in humans using functional imaging [7]. Although the current simulations focused on autoassociative function in the key region of CA3, interactions across multiple neural circuits are known to underlie cognitive behaviors like learning and memory. Nonetheless, much of the cortex is organized in recurrent circuits, and could process and store information in a sparser but analogous manner to CA3. Therefore, especially in cases contributed to by genetic disruptions with potentially widespread effects, the predicted manipulations aimed at rebalancing function could be broadly beneficial across neural networks underlying a pathologically extreme cognitive style. Ultimately tests of the types of predictions outlined in **Fig. **8 and detailed above, will support or refute this theorized approach of personalized cognitive therapeutics.

## Methods

### Network Simulations

Network simulations were constructed using NeuroConstruct [44] and simulations were run using Neuron [45]. Custom Matlab (Mathworks) scripts were used to generate stimulus pattern sets, calculate synaptic plasticity, and analyze simulation output. Each neuron was modeled as an isopotential sphere with a radius of 10 μm and had a membrane capacitance of 1.0 μF/cm^2 ^and contained a leak conductance with E_leak _= -67 mV (G_Leak _= 0.1 μS/cm^2^), and Fast Na^+ ^(G_Na _= 100 μS/cm^2^), and Delayed Rectifier K^+ ^(G_K _= 80 μS/cm^2^) conductances, based on a reduced model of hippocampal rhythm generation [15] (see **Fig. **2). Na^+ ^current was calculated as: I_Na _= G_Na_m^3^h(V_m_-E_Na_), with E_Na _= 90 mV, K^+ ^current was calculated as: I_K _= G_K_n^4^(V_m_-E_K_), with E_K _= -100. AMPA conductances of excitatory synapses had time courses described by G_AMPA _= exp^(-t/tau2) ^- exp^(-t/tau1)^, with tau1 = 1 and tau2 = 4, E_AMPA _= 0 mV, and GABA_A _conductances had time courses described by: G_GABA _= exp^(-t/tau2)^-exp^(-t/tau1)^, with tau1 = 2 and tau2 = 8, E_GABA _= -80 mV. Synaptic delays were 1 ms and axonal conduction times were considered negligible.

### Connectivity

For each simulation of a given connectivity level, three different connectivity profiles were generated using different random seeds in NeuroConstruct. The average performance of simulations using 10 different random sets of memories in each connectivity profile was calculated. Error rates are presented as mean ± SEM of the average performance in three connectivity profiles.

### Synaptic Plasticity

The weight of associational connections (W) following synaptic plasticity was determined using an adaptation of the equation used in the model of autoassociation we based our simulations on [13]: W_ij _= n_ij_^11^/(n_ij_^11^*γ^11^+n_ij_^10^*γ^10^+n_ij_^01^* γ^01^), where n_ij_^11 ^is the number of patterns where presynaptic(i) and postsynaptic(j) neurons fire together, and n_ij_^10 ^and n_ij_^01 ^are the number of patterns where presynaptic or postsynaptic neurons fire independently within a set of stored patterns. For simplicity in our implementation, γ^11 ^was set to a value of 1, and γ^10 ^and γ^01 ^shared the same value, γ^LTD^. This yielded the equation; W_ij_= g^Max^AMPA *n_ij_^11^/(n_ij_^11^+(n_ij_^10^+n_ij_^01^)*γ^LTD^), where the normalized strength of maximal potentiation, g^Max^AMPA, defines the strength of LTP, and the single parameter, γ^LTD^, defines the strength of LTD (**Fig. **3B).

### Inhibition

The strength of the excitatory synapses onto the inhibitory neuron was set to 90% of g^Max^AMPA. The strength of inhibitory synaptic conductances in the network was set relative to the maximal total excitation received by a pyramidal neuron during a stimulus pattern involving 10 neurons such that: gGABA = g^Max^AMPA*10*Connectivity Level*Relative Inhibition.

### Database Generation

Parameter permutations consisting of 20 connectivity levels spaced between 5 and 100%, 20 g^Max^AMPA values spaced between 2.78 and 55.56 nS, 20 logarithmically spaced Inhibition Ratio values between 0.01 and 100 (corresponding to 1.83 fold increments), and γLTD values logarithmically spaced between 0.1 and 10 (corresponding to 1.27 fold increments) were evaluated. For each of these 160,000 permutations of synaptic properties, 6 different sized pattern sets were assessed using multiple connectivity and stimulus pattern random seed conditions (see connectivity), for a total of 180 simulations per parameter combination. To facilitate the required large number of simulations, an approximation was made, based on the fact that the presence or absence of a spike in each individual neuron (which determines pattern storage or pattern completion success or failure), is determined entirely by, 1) the total excitatory conductance resulting from the properties of synaptic plasticity in the context of connectivity, and 2) the strength of inhibition. Therefore, a table of spike thresholds as a function of both total excitatory and inhibitory conductance received by a neuron was generated from a set of simulations with systematic variations in these properties. During creation of the database, pattern separation and pattern completion errors were assessed based on neuronal firing patterns determined by comparing values of total excitatory and inhibitory conductances in each neuron to the table of firing thresholds. Validation of this efficiency measure was performed by directly comparing several key parameter combinations using the threshold-table approximation with full explicit simulations. Optimal balanced networks were defined as the parameter combinations with the lowest maximal error (the higher error in either pattern separation or pattern completion), thus reflecting an even breakdown of pattern separation and completion for a given number of stored patterns.

## Competing interests

The authors declare that they have no competing interests.

## Authors' contributions

JEH conceived of the study, participated in the design of the study, carried out the computational simulations, participated in the interpretations of the results and drafted the manuscript. DVM participated in the design of the study and interpretation of the results and contributed to the intellectual content of the manuscript. All authors read and approved the final manuscript.

## Aknowledgements

This work was supported by grants from the National Institute of Mental Health (MH065541) and by The G. Harold and Leila Y. Mathers Charitable Foundation.
